# A Cross-Temporal Meta-Analysis on Marital Satisfaction of Chinese Couples

**DOI:** 10.3389/fpsyg.2022.903276

**Published:** 2022-06-29

**Authors:** Fengzhan Li, Chen Chen, Jinrui Wang, Haiyun Peng, Lin Wu, Lei Ren, Lei Song, Yinchuan Jin, Qun Yang

**Affiliations:** ^1^Department of Military Medical Psychology, Air Force Medical University, Chinese People's Liberation Army (PLA), Xi'an, China; ^2^Department of Psychology, School of Education Science, Ludong University, Yantai, China

**Keywords:** couples, marital satisfaction, social changes, cross-temporal meta-analysis, cultural environments

## Abstract

**Objective:**

To investigate the changing trend of Chinese couples' marital satisfaction and its relationship with social changes.

**Methods:**

A cross-temporal meta-analysis was performed on 118 original studies (*n* = 31,909) reporting marital satisfaction of Chinese couples from 1994 to 2020, primarily using correlation analysis and regression analysis.

**Results:**

(1) Overall, the marital satisfaction of Chinese couples showed a downward trend over time. (2) Men's marital satisfaction displayed almost no change, while women's marital satisfaction had a more obvious downward trend. (3) Changes in macrosocial factors (per capita consumption expenditure, housing prices, old-age dependency ratio, and divorce rate) could significantly predict the downward trend of marital satisfaction, especially for women.

**Conclusion:**

In the past 27 years, the overall marital satisfaction level of Chinese couples has shown a downward trend, and there are gendered differences, which may be related to changes in the socioeconomic and cultural environments.

## Introduction

Marriage is the longest, most intimate and most unique interpersonal relationship in life (Levenson et al., 1993) and is an important source of happiness in people's lives. It is a prerequisite for the stability of a marriage to create satisfaction in the marital relationship. Marital satisfaction refers to an individual's attitudes and views on spouses and marital relationships (Hamilton, 1929), which is considered to be a key factor in marital quality (Li and Fung, 2011) and an important path that affects marital outcomes (Karney and Bradbury, 1995). Chapman and Guven (2016) point out that the subjective wellbeing of individuals is affected by marital satisfaction, and people with poor marital satisfaction are less happy than unmarried people. Marital satisfaction not only has a significant impact on the couple's work and life but also affects their physical and mental health and even their children (O'Connor et al., 1999; Davila et al., 2003; Pruchno et al., 2009). Therefore, it is of great significance to study couples' marital satisfaction.

### Research Status of Chinese Couples' Marital Satisfaction

Studies on marital satisfaction in China began in the 1990s. Some studies found that Chinese couples were relatively satisfied with their marriages (Wang and Yu, 2013), but other studies showed that trends in the marital satisfaction of Chinese couples was not optimistic (Wang et al., 1997). On the one hand, the reason for the inconsistency in conclusions may be because the subjects came from different regions, and the economic development levels of these regions were quite different. A study found that the level of economic development could affect marital satisfaction (Tavakol et al., 2017). On the other hand, the more important reason is that the existing studies are all cross-sectional studies. In other words, the studies focused on the marital satisfaction of couples in a certain period or a certain age group but neglected its potential changes over time.

In addition, many studies on the influencing factors of marital satisfaction have focused on individual and family demographics, such as gender, age, education level, work status, coping style, social support, family income, domestic violence, and communication styles (Rosen Grandon et al., 2004; Jose and Alfons, 2007; Ng et al., 2009; Fuenfhausen and Cashwell, 2013; Richter et al., 2015; Liu et al., 2016; Ghahremani et al., 2017; Tavakol et al., 2017; Dobrowolska et al., 2020; Karney, 2021). Few studies have paid attention to the impact of changes in macrosocial factors on marital satisfaction. According to the ecosystem theory, individual psychological development is nested in a series of mutually influencing environmental systems, in which the systems interact with the individuals and influence them. The systems include microscopic systems, intermediate systems, outer systems and macrosystems. The macrosystem refers to the cultural and social environments, both of which could affect the individual's mental state (Bronfenbrenner, 1994). Over the past 40 years of reform and opening, China has made remarkable economic, social, and cultural achievements. While the mode of production and lifestyles have undergone tremendous changes, some negative social problems have also emerged. For example, some studies have found that the happiness of Chinese teachers was on the decline (Xin et al., 2021), the work-life imbalance in the workplace was on the rise (Xin et al., 2020), and the mental health status of nurses was on the decline (Xin et al., 2019). These psychological variables (happiness, work-life balance, mental health) were also closely related to the level of marital satisfaction (Glenn, 1975; Diener et al., 2000). Does the marital satisfaction of Chinese couples also show a downward trend with socioeconomic and cultural environmental changes? The present study will answer this question by the method of cross-temporal meta-analysis.

### The Cross-Temporal Meta-Analysis

The cross-temporal meta-analysis was first used in an empirical study by the American scholar Twenge (1997). The basic principle of this method is to find coherency among isolated existing studies in chronological order, making these studies a cross-sectional sampling of historical development. With this method, it is possible to analyze the changes to psychological variables related to a large time span from a macroscopic perspective. This method can reveal the changing trend of psychological variables over time and can also reveal how social changes affect individual psychological development by analyzing the relationship between psychological variables and social indicators (Xin and Chi, 2008). Twenge adopted this approach to examine changes in various psychological variables over time, such as anxiety, self-esteem, and wellbeing (Twenge, 2000; Twenge and Campbell, 2001; Twenge et al., 2016). In China, Xin and Chi (2008) first introduced this method in detail, and then a large number of scholars carried out a series of studies using this method, all of which found that social changes affected people's psychological status (Xin and Zhang, 2009; Xin et al., 2011; Yang et al., 2014; Su and Liu, 2020, 2021).

### Social Indicators

At present, although China has become the second largest economy in the world and its GDP has been rising over the years, the consumption level of residents and housing prices are also rising. In addition to socioeconomic changes, there are also some other important changes, such as a rising divorce rate and an increasingly serious rise in the elderly population (Du and Li, 2021), which closely impact many families. Specifically, the rise in household consumption levels and housing prices will inevitably bring greater economic pressure to Chinese couples. The increase in the divorce rate means that family stability is widely perceived as threatened, which may aggravate couples' sense of insecurity about their own marital relationship. The aging population means that Chinese couples need to devote more time and energy to supporting their elders.

Therefore, the present study selected per capita household consumption expenditures, housing prices, the old-age dependency ratio (referring to the ratio of the population aged 65 and above to the working-age population), and the divorce rate (the ratio of divorces within 1 year to the total population) as the indicators of change in the social environment to examine the impact on marital satisfaction. The first two indicators reflect the changes in economic development. The old-age dependency ratio reflects the situation of Chinese couples supporting the elderly; the larger the value is, the more elderly people Chinese couples need to support on average. The rising divorce rate reflects changes in the cultural concept of marriage. It is worth mentioning that these four social indicators were also had been used to measure social changes in other similar studies (Xin et al., 2020, 2021).

In summary, the present study adopts cross-temporal meta-analysis to establish the trend in Chinese couples' marital satisfaction over time and reveals the change in the mean scores of marital satisfaction from a longitudinal perspective. At the same time, this study reveals the impact of socioeconomic and cultural environment changes on marital satisfaction by examining the relationship between representative social indicators and marital satisfaction.

## Methods

### Literature Search

Original studies about marital satisfaction were searched for in the following three Chinese academic literature databases in January 2022: China National Knowledge Internet, Wanfang and Chongqing VIP Information. The three databases include almost all periodicals in science and social science, including those with psychology themes and other similar subjects. The full-text search was carried out with terms such as “marital satisfaction,” “marital quality,” “marital status,” and “marriage happiness.” Additionally, the same procedure was used to retrieve English language literature from the databases PubMed, Medline, PsycINFO, Web of Science, Elsevier, Wiley and PsycArticles.

### Inclusion Criteria

The first point that needs to be noted is that the present study only focused on the literature used the marital satisfaction subscale of the Olson Marital Quality Questionnaire because its Chinese version has good reliability and validity (Li and Yang, 1990; Wang et al., 1999) and is already one of the most widely used scale in China. This questionnaire was first established by Fowers and Olson (1989) and contains 12 subscales. The marital satisfaction subscale is one of them and is used to measure the individual's degree of satisfaction with the marital relationship (Fowers and Olson, 1993). Its Chinese version contains 10 items and uses a 5-point scale, for a maximum score of 50. The higher the score is, the more harmonious and satisfactory the marital relationship is considered to be in most aspects. Therefore, the literature included in the meta-analysis needed to adopt the marital satisfaction subscale of the Olson Marital Quality Questionnaire.

**Figure 1:**
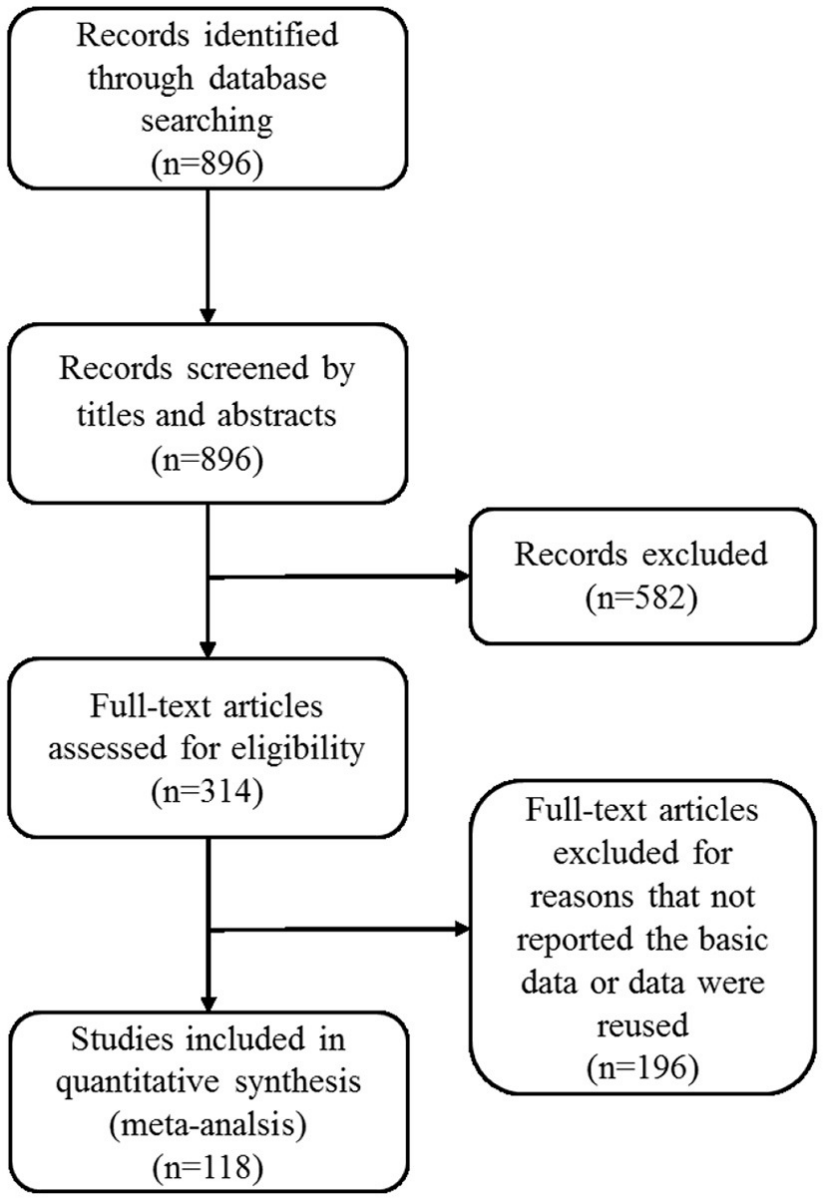
PRISMA flow chart for the screening of studies.

In addition, the included literature also needed to meet the following conditions: (1) the subjects were Chinese mainland couples under the age of 60; (2) the mean scores and standard deviations of marital satisfaction were reported; and (3) the earliest study was selected when the same dataset was issued two or more times. The included studies met all of the above criteria; otherwise, they were excluded. Finally, one hundred eighteen articles were finally adopted for further analysis, including a total of 31,909 subjects. To illustrate the inclusion strategy and the results of the search strategy, a PRISMA diagram is depicted in Figure 1.

Since there were no articles that met the above criteria before 1996, all the included articles were published between 1996 and 2021. According to the practice of previous studies (Twenge, 2000; Xin and Zhang, 2009), the collection year (referred to as “Year”) of the articles was calculated by subtracting 2 years from the publication year, except for the articles pointing out the specific collection year. Therefore, the period of the present study is 1994 to 2020, for a total of 27 years. The details of the data are shown in Table 1.

**Table 1:** The number and distribution of Chinese couples' marital satisfaction data.

**Years of**	**Number of**	**Sample**	**Gender**	
**data collection**	**datasets**	**size**	
			**Number of**	**Number of**
			**male groups**	**female groups**
1994	1	104	1	1
1995	1	52	0	1
1996	2	674	0	0
1997	2	228	1	1
1998	1	137	0	1
1999	5	234	1	1
2000	4	1,179	1	3
2001	5	466	0	3
2002	2	121	0	1
2003	1	60	1	0
2004	8	3,120	2	4
2005	8	1,056	1	3
2006	10	4,125	1	6
2007	5	1,663	2	3
2008	7	2,358	1	5
2009	4	793	1	4
2010	4	448	0	3
2011	6	1,887	1	2
2012	7	2,155	4	4
2013	4	969	0	4
2014	1	290	0	1
2015	6	1,934	3	3
2016	6	2,801	2	1
2017	7	1,778	2	3
2018	5	1,392	2	3
2019	4	1,229	3	3
2020	2	656	2	2
Total	118	31,909	32	66

### Control Variables

Given regional distinctions and the quality standards of publications, these variables were likely to differ across the reported data. According to Xin and Zhang (2009), the types of publications and the regions from which the participants came were controlled by classifying them into different categories as follows: for publications, 1 = core journal, 2 = general journal, 3 = thesis or dissertation; for regions, 0 = no clear regional information, 1 = eastern region, 2 = northeast region, 3 = central region, 4 = western region, 5 = covering multiple regions.

### Sources of Social Indicator Data

The present study selects “per capita household consumption expenditure,” “housing price,” “old-age dependency ratio,” and “divorce rate” as social indicators to measure China's socioeconomic and cultural environment. The data for the first three social indicators come from the China Statistical Yearbook. The divorce rate data come from the China Civil Affairs Statistical Yearbook.

### Data Analysis Strategy

At first, it should be noted that, in traditional meta-analysis, it focuses on an effect size for each study and need use the comprehensive meta-analysis. However, the cross-temporal meta-analysis aims at investigating the change in mean scores on psychological measures over time mainly by correlation analysis and regression analysis, which is different from traditional meta-analysis. In the present study, the correlation analysis and regression analysis were conducted between marital satisfaction mean scores of each study and year of data collection.

Then, a weighted regression equation and the average standard deviation (SD) of the individual samples were adopted to calculate the magnitude of the changes in marital satisfaction scores. In present study, the calculation formula is $d=\frac{M_{2020}-M_{1994}}{M_{SD}}$. Firstly, a regression equation was used to calculate the mean scores for a specific year (i.e., *M*_2020_ and *M*_1994_): *Y* = b *X* + c, where b = the partial regression coefficient, c = the intercept or constant, *X* = the year of data collection, and *Y* = the predicted mean score of marital satisfaction. This regression equation could yield the expected average marital satisfaction scores for 2020 and 1994. Secondly, the average SD (i.e., *M*_SD_) was computed by averaging the within-sample SDs reported in the studies. It is important to note that this method of calculation is likely to avoid the ecological fallacy (Rosenthal et al., 2000), which occurs when the magnitude of change is calculated using the variation in mean scores, it exaggerates the change magnitude because mean scores are not differ as much as individual scores. Therefore, the variation within a population of individuals should be used to calculate the magnitude of change, that is, we use the average SD of the individual studies to capture the variance of the marital satisfaction scale among Chinese couples.

Finally, we performed a time lag analysis to explore whether social indicators could explain changes in marital satisfaction or not. The social indicators of each year was matched with the marital satisfaction mean score of each study in two ways: the year of data collection, and 3 years before the data were collected. For instance, a data point for marital satisfaction score of each study from 2020 was matched with each social indicators from 2017, 2020. According to Twenge (2000), if social indicators could explain the changes in marital satisfaction over time, the correlations should be significant when marital satisfaction scores are matched with social indicators at several years before. Furthermore, if marital satisfaction and social indicators have concurrent relationships, the correlations should be significant when marital satisfaction scores are matched with social indicators at the year of data collection.

## Results

### Changes in Marital Satisfaction Over Time

**Figure 2:**
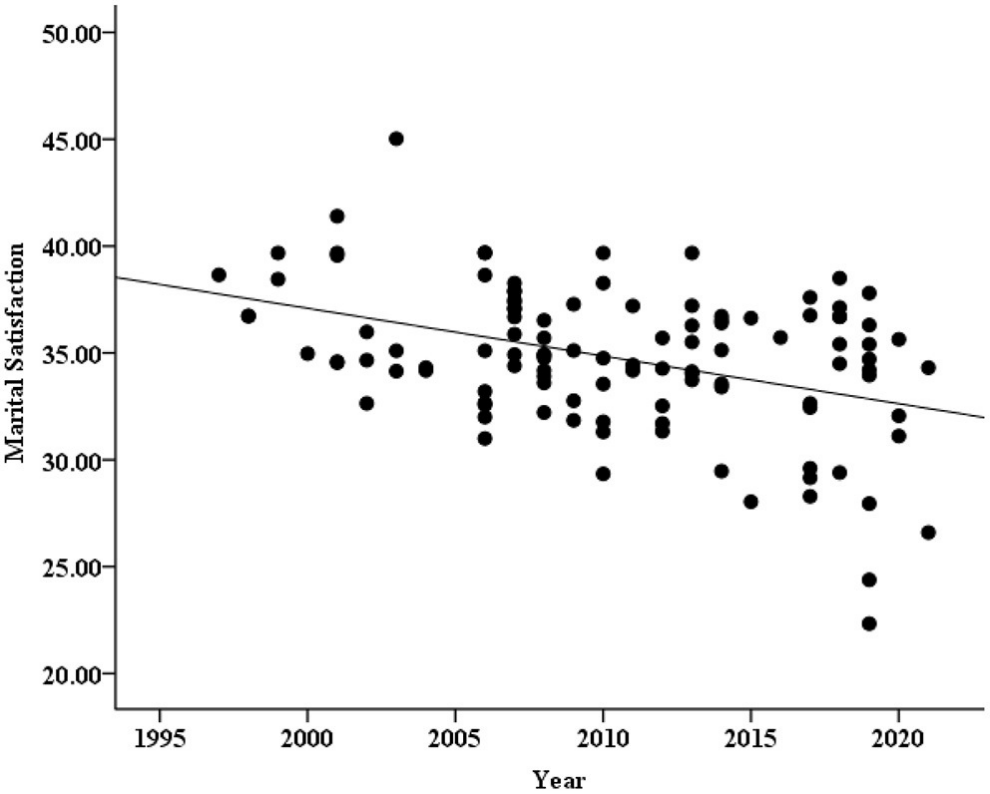
Scatter plot between marital satisfaction and years.

A scatter plot between Chinese couples' marital satisfaction and years was created to examine the relationship between them, and it was found that the mean scores of marital satisfaction decreased over time (see Figure 2).

To accurately quantify and describe the changes in marital satisfaction over time, correlation analysis was carried out between the mean scores of marital satisfaction and year according to the data analysis method of previous researchers (Twenge and Im, 2007; Xin and Zhang, 2009). The results showed that marital satisfaction was significantly negatively correlated with the passage of time (*r* = −0.40, *p* < 0.001). In the studies of cross-temporal meta-analysis, the results are highly susceptible to confounding by the sample size of each article, journal type, and other factors. Therefore, according to previous studies, a regression analysis on the basis of weighted sample size was carried out between the mean scores of marital satisfaction (as the predicted variable) and year (as the predictor variable) with the journal type and region as the control variables. It was found that the year could significantly negatively predict the changes in marital satisfaction [β = −0.32, *SE* = 0.05, *r*^2^ = 0.14, *p* = 0.001, 95% CI = (−0.26, −0.07)] after controlling for these variables. In short, it can be seen that the overall marital satisfaction of Chinese couples showed a downward trend from 1994 to 2020.

### Magnitude of Changes in Marital Satisfaction

According to previous studies (Twenge and Im, 2007; Xin and Zhang, 2009), the regression equation and the average standard deviation of all the samples were used for analysis, and the effect size (*d*) was calculated to measure the magnitude of change in marital satisfaction in the past 27 years. First, we established a regression equation (*Y*_MS_ = −0.168*X*_year_ + 372.027, where *Y*_MS_ is the mean score of marital satisfaction, −0.168 is the partial regression coefficient, and *X*_year_ is the year of data collection) on the basis of weighting the sample size. Second, 1994 and 2020 were substituted into the regression equation to obtain the average scores of the 2 years: *M*_1994_ = 37.035 (the average score of the starting year) and *M*_2020_ = 32.667 (the average score of the ending year). Finally, the difference between *M*_2020_ and *M*_1994_ was calculated and divided by the 27-year mean standard deviation *M*_*SD*_ (average score of standard deviations across all studies) to obtain the *d* value.

The results showed that the marital satisfaction score of Chinese couples dropped by 4.368 points from 1994 to 2020, which was a drop of 0.74 standard deviations (*d* = −0.74). Cohen considered an effect size (absolute value) between 0.2 and 0.5 to be a “small effect”, between 0.5 and 0.8 to be a “moderate effect” (an effect sufficient to attract attention), and >0.8 to be a “large effect” (Cohen, 1988). According to this criterion, the decrease in marital satisfaction achieved a “moderate effect” size. In summary, the overall marital satisfaction level of Chinese couples has gradually declined over the past 27 years, with a moderate effect.

### Gender Differences in Changes in Marital Satisfaction Over Time

**Figure 3:**
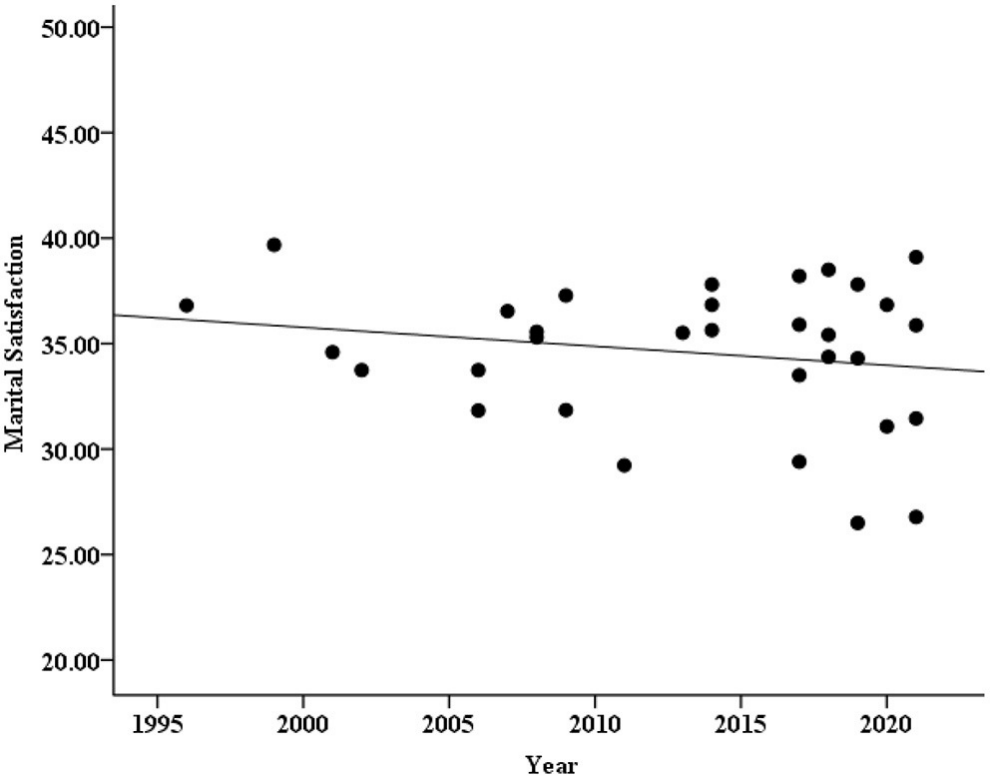
Scatter plot of male marital satisfaction and years.

**Figure 4:**
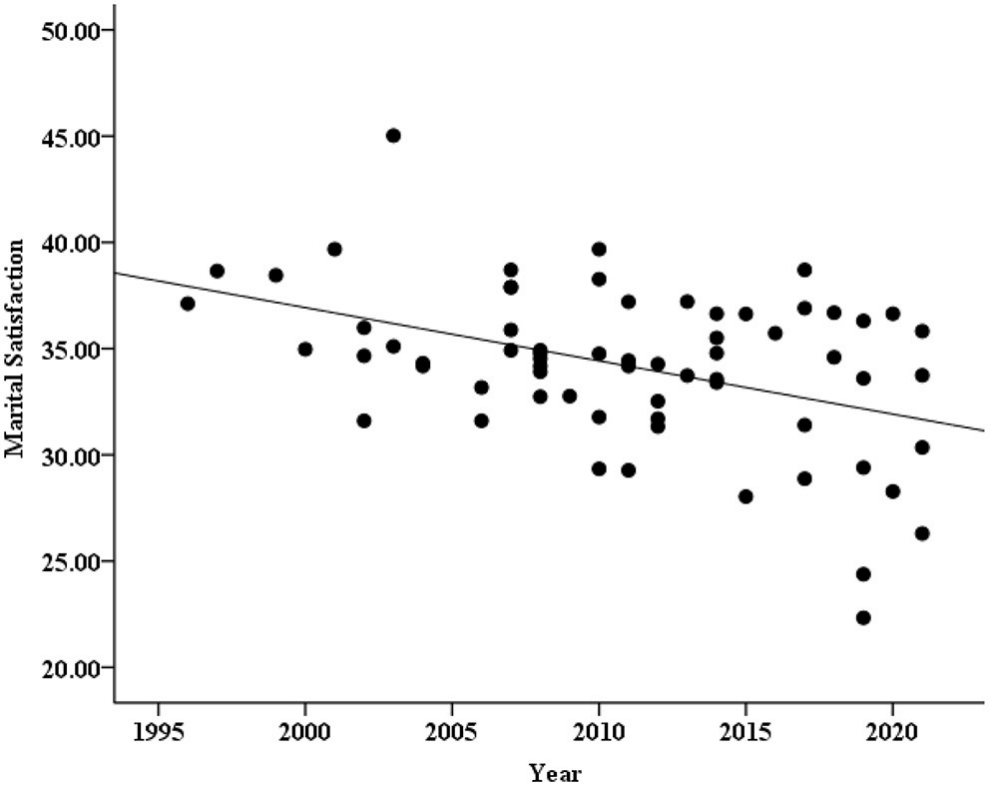
Scatter plot of female marital satisfaction and years.

As seen from Tables 1, 32 groups and 66 groups of data reported the marital satisfaction of men and women, respectively, but other articles reported only the overall marital satisfaction. To explore the changing trend of marital satisfaction in different genders, we drew two scatter plots (see Figures 3, 4) and carried out the correlation analysis. The results found that male marital satisfaction barely changed over time (*r* = −0.20, *p* = 0.28), but female marital satisfaction showed an obvious downward trend (*r* = −0.45, *p* < 0.001).

Similarly, according to the above method, regression analysis was carried out on marital satisfaction and year for different genders after weighting the sample size with the journal type and region as the control variables. It was found that the year could significantly predict the level of female marital satisfaction, and the year explanation rate was 16% [β = −0.40, *SE* = 0.06, *r*^2^ = 0.16, *p* = 0.001, 95% CI = (−0.34, −0.09)], while the prediction for male marital satisfaction was not significant [β = −0.04, *SE* = 0.11, *r*^2^ = 0.07, *p* = 0.86, 95% CI = (−0.25, 0.21)]. It can be concluded that in general, men's marital satisfaction did not change significantly over time, while women's marital satisfaction showed a downward trend over time.

To quantify the changes in marital satisfaction of different genders over time in more detail, we calculated the effect size *d*. The result showed and the effect of changes in male marital satisfaction was under a “small effect” size (*d* = −0.09, which could be ignored), while the decrease in female marital satisfaction was achieved a “large effect” size (*d* = −0.93). In addition, as a reviewer's suggestion, we also conducted a difference test between male and female standardized regression coefficients to make the conclusion more reliable. Since both the regression equation between marital satisfaction and year for men and women are ultimately linear regression model (the journal type and region which as control variables fail to enter in the regression equation, and the sample sizes were weighted), the difference test between two standard regression coefficient is actually test the difference between two correlation coefficients (i.e., the value of *r* is equal to β). Therefore, the formula $Z=\frac{Z_{r_{1}}-Z_{r_{2}}}{\sqrt{\frac{1}{n_{1}-3}+\frac{1}{n_{2}-3}}}$ could be used for the difference test (*n*_1_ = 66, namely 66 groups of data reported the marital satisfaction of women; *n*_2_ = 32, namely 32 groups of data reported the marital satisfaction of men; *r*_1_ = −0.40, *r*_2_ = −0.04). The result showed that the standardized regression coefficient of women was significantly greater than that of men (*Z* = −1.87, *p* = 0.031). Based on the above results, it can be concluded that there is almost no change in male marital satisfaction, while the downward trend and magnitude of female marital satisfaction is very obvious.

### Relationship Between Marital Satisfaction and Social Indicators

Is there a relationship between the gradual decline in marital satisfaction of Chinese couples and social changes from 1994 to 2020? As mentioned above, the correlation analysis between the mean scores of marital satisfaction and social indicators could answer this question. The results of the correlation analysis are shown in Table 2. After controlling for the sample size, the variables “per capita household consumption expenditure,” “housing price,” “old-age dependency ratio,” and “divorce rate” were all significantly negatively correlated with marital satisfaction on the whole. Analysis of gender differences revealed that the correlations between social indicators and marital satisfaction were all non-significant for men but all significant for women. In addition, the direction of correlations between female marital satisfaction and social indicators was consistent with overall trends, and the correlation strengths of the former were all stronger than those of the latter.

**Table 2:** The correlations between social indicators and marital satisfaction.

**Social indicators**	**MS of total**	**MS of men**	**MS of women**
**The year of data collection**
Per capita household consumption expenditure	−0.38^**^	−0.19	−0.43^**^
Housing price	−0.38^**^	−0.21	−0.44^**^
Old-age dependency ratio	−0.39^**^	−0.24	−0.44^**^
Divorce rate	−0.38^**^	−0.14	−0.44^**^
**Three years before data collection**
Per capita household consumption expenditure	−0.39^**^	−0.21	−0.44^**^
Housing price	−0.38^**^	−0.19	−0.43^**^
Old-age dependency ratio	−0.39^**^	−0.23	−0.44^**^
Divorce rate	−0.37^**^	−0.18	−0.44^**^

** *p < 0.01*.

To further illustrate the relationship between socioeconomic and cultural environment factors and changes in marital satisfaction, lag correlation analysis was carried out according to the practice of previous studies (Xin et al., 2010, 2011), that is, the mean scores of marital satisfaction were matched and correlated with social indicators from 3 years before. The results found that the correlations between marital satisfaction and the social indicators from 3 years before were basically consistent with the correlations for the year of data collection, whether overall or for the male and female groups. The correlation strength for the female groups was stronger than that of the overall group. Based on the above results, these social indicators may have “influenced” the changes in the marital satisfaction of Chinese couples, especially for women.

## Discussion

### The Changing Trend of Chinese Couples' Marital Satisfaction

The present study adopted a cross-temporal meta-analysis to examine the changing trends of Chinese couples' marital satisfaction from 1994 to 2020. The results found that the level of Chinese couples' marital satisfaction generally showed a downward trend, and the magnitude of changes in mean scores dropped by 0.74 standard deviations in the past 27 years, which was basically consistent with the views of some scholars on the decrease in Chinese couples' marital satisfaction (Xu, 1991; Wang et al., 1997) and resolved the controversy of previous studies to a certain extent. However, the difference from the previous cross-sectional studies is as follows: First, the present study adds the time dimension to examine the changing trend of Chinese couples' marital satisfaction and stretches the previous empirical studies on this psychological variable from a longitudinal perspective. Second, the previous studies focused on the individual's marital satisfaction, and the generalization of the conclusions obtained was actually limited, while the present study focused on the group by adopting the method of cross-temporal meta-analysis. The analysis of 118 research data points can make the results of the present study better reflect the status of Chinese couples' marital satisfaction and expand the breadth of the understanding of group psychological variables. In short, the present study examines the changes in marital satisfaction of Chinese couples from a longitudinal perspective, which makes the research conclusions more abundant and reliable.

In addition, further data analysis found that the trend of declining marital satisfaction was more obviously in women, but there was almost no change in male marital satisfaction. In the past 27 years, the magnitude of change in the level of male marital satisfaction was only a standard deviation of 0.19, while female marital satisfaction dropped by a standard deviation of 0.94. This result may reflect gender differences in marital satisfaction. In fact, the phenomenon that men and women perceive differently in marriage is very common in related researches. For example, in 1972, prominent family scholar Jesse Bernard famously stated, “There are two marriages in every marital union, his and hers. And his…is better than hers” (Bernard, 1972). Many studies have confirmed this phenomenon and also found that Chinese women generally have lower marital satisfaction than men (Shek, 1995; Lu, 2006; Ng et al., 2009). Bernard (1972) believed that there were two reasons for this phenomenon. One reason was that men have fewer emotional needs, while women have higher expectations for marriage, and high expectations are more likely to lead to a stronger psychological discrepancy. Another explanation was that the division of labor differs between men and women. Generally, men are usually responsible for primary income, and women are responsible for taking care of children and families, which inevitably leads to conflicts between work and family (Ferree, 1990). Although the above explanations for gender differences in marital satisfaction didn't take into account the changes of time, the phenomenon they described has become more and more obvious in Chinese society (Zhu, 2011; Chang, 2020; Yu and Liu, 2021). Therefore, the obvious downward trend of women's marital satisfaction may be related to their increasing high marital expectations and work-family conflicts.

To sum up, the presesnt study provides a new perspective on understanding gender differences in marital satisfaction. Although numerous studies have shown that men were generally more satisfied with their marriages than women (Shek, 1995; Lu, 2006; Zhang et al., 2012), they were all based on cross-sectional studies. However, the present study found that Chinese men's marital satisfaction is stable, while women's marital satisfaction has a downward trend based on a longitudinal research perspective. This finding enriched the studies on gender differences in marital satisfaction.

### The Impact of Social Changes on Marital Satisfaction

Based on the ecosystem theory, the present study analyzes the influencing factors of changes in couples' marital satisfaction at the macrosocial level.

First, the decline in marital satisfaction of Chinese couples may be related to the economic pressures they face. The present study found that the socioeconomic indicators for 3 years before and the year of data collection (per capita household consumption expenditure, housing prices) could significantly negatively predict marital satisfaction, and this prediction effect was more obvious for female groups. These results suggested that the economic pressures faced by Chinese couples have a negative impact on marital satisfaction, which is consistent with existing research (Vaijayanthimala et al., 2004; Archuleta et al., 2011). At present, although the level of China's economic development is improving year by year, living costs such as the prices of commodities and housing are also on the rise. In the past two decades, China's housing prices have almost increased by a factor of 10, which has brought huge and direct economic pressures to Chinese couples. Couples need more income to cope with these economic changes, but achieving more income means that they must devote more time and energy to work, which will inevitably lead to conflict between work and family as well as a decline in marital satisfaction (Liu et al., 2016). In addition, the present study found that the decline in marital satisfaction is mainly reflected in women. On the one hand, the reason for this is that there are gender differences in marital satisfaction. On the other hand, it may also be related to the rise in housing prices. Studies have found that the higher the housing price is, the more negative the impact on married life (Yu and Zhou, 2015), especially for Chinese women, because they usually pay more attention to housing issues (Zhao et al., 2019). That is, many families may not be able to afford housing with rising prices, which affects the quality of life in turn and leads to a decline in women's marital satisfaction. This conclusion suggests that the Chinese government could take measures such as stabilizing the prices of commodities and housing to help Chinese couples improve their marital satisfaction.

Second, the decline in marital satisfaction of Chinese couples may be related to the burden of supporting the elderly. For Chinese society, “filial piety” has always been the focus of Chinese social culture. It is an important obligation of Chinese couples to support the elderly. The data show that the aging of Chinese society is becoming increasingly serious. The old-age dependency ratio reflects the status of Chinese couples supporting the elderly; that is, the larger the value is, the more elderly people Chinese couples need to support on average. In short, the burden of supporting the elderly is getting heavier for Chinese couples. The present study found that the more elderly people there are to support in the family, the lower couples' marital satisfaction level. This is basically consistent with the views of Zeng and Zou (2017). They believed that supporting the elderly requires many material, spiritual, psychological and emotional costs from husbands and wives. The rising cost of living and fierce pressure of competition lead to physical and mental exhaustion and lack of meaning in life (Zeng and Zou, 2017). Therefore, the couple's marital satisfaction would be affected naturally. In addition, there is a traditional idea of “men outside; women inside” in Chinese families, which leads to the fact that women usually spend more time and energy supporting the elderly. Furthermore, many women also need to participate in work under economic pressure. This means that women suffer from the dual stress of work and family care (Choi, 2008). Therefore, the old-age dependency ratio could significantly negatively predict the decline in female marital satisfaction. This conclusion suggests that the government could also adopt the method of improving old-age care and providing old-age services for the improvement of marital satisfaction.

Finally, the decline in Chinese couples' marital satisfaction may also be related to changes in social-cultural beliefs about marriage. In fact, the values of marriage and family life in Chinese social culture have also been strongly impacted by economic development. In traditional culture, Chinese society has a negative attitude toward divorce. However, data show that the divorce rate of Chinese couples is increasing, which reflects the changes in the cultural valuation of marriage to a certain extent; that is, divorce has increasingly become a way of life and a free personal choice (Dong, 2016). This marriage culture embodies social progress but may cause Chinese couples to feel uneasy about the stability of the family at the same time. The present study shows that the divorce rate in Chinese society could significantly predict the decline in marital satisfaction of couples, but this phenomenon is mainly reflected in the female group. This is consistent with other studies that showed that women are more susceptible to social environmental influence and are more likely to perceive stress (Fox et al., 2014; Seo et al., 2017). These characteristics would lead to a more likely decline in female marital satisfaction (Ledermann et al., 2010). This conclusion suggests that the Chinese government may need to promote some more positive marriage values in society.

Therefore, it could be seen from the above that, with the changes of the times, the representative macro factors that reflect the social development status (such as socioeconomic conditions, burden of providing for the elderly and cultural atmosphere) are important and cannot be ignored in predicting the specific psychological variables of some groups (such as the marital satisfaction of couples in the present study). The results of studies obtained by using cross-temporal meta-analysis could not only focus on macrosocial indicators related to the psychological variables but also provide more accurate ideas for policies targeting adjustments to specific psychological variables, which is more important. At the same time, the results could provide new evidence for ecological system theories.

### Limitations

The present study has the following limitations. First, it does not distinguish the marital satisfaction of couples of different ages because couples of different ages may have different perceptions of marriage. More detailed studies can be carried out in the future. Second, the causal relationship between social changes and marital satisfaction needs to be further examined by longitudinal studies, and its internal impact mechanism also needs to be explored. Finally, the effectiveness of the cross-temporal meta-analysis method in reducing ecological fallacy was challenged by some researchers (Trzesniewski and Donnellan, 2010), and it has also been criticized for systematically overestimating effect sizes (Rudolph et al., 2020). Thus, we recommend that future research verify the reliability of these results of the present study using the method proposed by Trzesniewski and Donnellan (2010) and Rudolph et al. (2020).

## Conclusion

In conclusion, the present study found that the overall marital satisfaction of Chinese couples has shown a downward trend in the past 27 years, especially for women. Based on ecosystem theory, this downward trend may be related to economic pressure and the burden of providing for the elderly on the one hand and the change in marriage culture on the other hand. These findings may have some implications for the Chinese government.

## Data Availability Statement

The original contributions presented in the study are included in the article/supplementary material, further inquiries can be directed to the corresponding author.

## Author Contributions

FL: idea and introduction. CC: method, results, and coding. JW and HP: discussing and coding. LW, LR, LS, and YJ: coding and revising language. QY: method, coding, and revising language. All authors contributed to the article and approved the submitted version.

## Funding

This paper was funded by Chinese Air Force Logistics Department (Grant Number: BKJ20J002).

## Conflict of Interest

The authors declare that the research was conducted in the absence of any commercial or financial relationships that could be construed as a potential conflict of interest.

## Publisher's Note

All claims expressed in this article are solely those of the authors and do not necessarily represent those of their affiliated organizations, or those of the publisher, the editors and the reviewers. Any product that may be evaluated in this article, or claim that may be made by its manufacturer, is not guaranteed or endorsed by the publisher.
